# Early detection of non-invasive ventilation failure among acute respiratory failure patients in the emergency department

**DOI:** 10.1186/s12873-020-00376-1

**Published:** 2020-10-07

**Authors:** W. Liengswangwong, C. Yuksen, T. Thepkong, P. Nakasint, C. Jenpanitpong

**Affiliations:** Department of Emergency Medicine, Faculty of Medicine, Ramathibodi Hospital, Mahidol University, 270, Rama 6 Road, Phayathai, Ratchathewi, Bangkok, 10400 Thailand

**Keywords:** NIV failure, Predictive score

## Abstract

**Background:**

Non-invasive mechanical ventilation (NIV) has become an alternative to an invasive artificial airway for the management of acute respiratory failure (ARF). NIV failure causes delayed intubation, which eventually has been associated with increased morbidity and mortality. This study aimed to develop the clinical scoring system of NIV failure in ARF patients.

**Methods:**

This study was a diagnostic, retrospectively cross-sectional, and exploratory model at the Emergency Medicine Department in Ramathibodi Hospital between February 2017 and December 2017. We included all of the acute respiratory failure patients aged > 18 years and received non-invasive ventilation (NIV). Clinical factors associated with NIV failure were recorded. The predictive model and prediction score for NIV failure were developed by multivariable logistic regression analysis.

**Result:**

A total of 329 acute respiratory failure patients have received NIV success (*N* = 237) and failure (*N* = 92). This study showed that NIV failure was associated with heart rate >  110 bpm, systolic BP <  110 mmHg, SpO2 < 90%, arterial pH < 7.30 and serum lactate. The clinical scores were classified into three groups: low, moderate, and high.

**Conclusion:**

We suggested that the novel clinical scoring of the NIV failure in this study may use as a good predictor for NIV failure in the emergency room.

## Background

Acute respiratory failure (ARF) was a steady increase in the number of hospitalizations at an average annual rate of 11.3% in 2001 to 2009 with a decrease in inpatient mortality in the United States [1]. In Thailand, ARF was increased from 6.99 people per 100,000 in 2011 to 8.98 people per 100,000 in 2014 [2]. ARF characterized by the impaired respiratory system to exchange gases and to oxygenate the blood, resulting in hypoxia with or without hypercapnia [2]. Two main mechanisms of ARF include failure in pulmonary ventilation caused by neuromuscular diseases, chest wall deformities, obstructive pulmonary diseases, and failure in gas exchanges caused by adult acute respiratory distress syndrome, neonatal respiratory distress syndrome, acute cardiogenic pulmonary edema, severe status asthmaticus, pneumonia, airspace collapse (atelectasis) and pulmonary embolism [3]. The clinical signs and symptoms of patients with ARF refer to the two main manifestations of pulmonary diseases, including arterial hypercapnia and hypoxemia.

Noninvasive ventilation (NIV) refers to the delivery of mechanical ventilation without using an invasive artificial airway (endotracheal tube or tracheostomy tube) that markedly increases over the past two decades worldwide [4]. NIV has become an alternative to orotracheal intubation and invasive mechanical ventilation for the management of ARF, since it can decrease the length of stay in the ICU, reduce the number of possible complications, increase the quality of life, reduce risk of infection and improve the chance of survival, compared to conventional invasive ventilation [5–7]. The effectiveness of NIV varies according to the etiology of respiratory failure [8]. However, NIV failure causes delayed intubation and was associated with an increased risk of in-hospital death, ICU and hospital stay [9]. Thus, early prediction of NIV failure is important. However, the clinical scoring system is lacking. Therefore, this study aimed to develop the clinical scoring system of NIV failure in ARF patients at the Emergency Medicine Department of Ramathibodi Hospital, a Mahidol university-affiliated super tertiary care hospital in Bangkok, Thailand.

## Method

This study was retrospectively cross-sectional study. Data was collected from Ramathibodi hospital database via Electronic Medical Record (EMR) by using NIV protocol record form between February 2017 and December 2017.

We included all acute respiratory failure patients aged > 18 years and received non-invasive ventilation (NIV) in the study period. We excluded the patients with denied intubation, used a tracheostomy tube, owned a personal non-invasive ventilator, and used non-invasive ventilation post-extubation.

The study variables were recorded for all eligible patients, including the baseline characteristic factor and potential clinical factors for NIV failure. Clinical factors included gender, age, vital signs at ED arrival (respiratory rate, heart rate, systolic blood pressure, oxygen saturation, body temperature), Glasgow coma scale, diagnosis, underlying disease, laboratory test, arterial blood gas, vasoactive agents and qSOFA score.

The outcomes were NIV success (did not receive intubation in this hospital admission) and the NIV failure (receive intubation in this hospital admission). Finally, we develop clinical scoring of failure on acute respiratory failure patients received NIV at the emergency department.

### Study size estimation

We collected the data of the acute respiratory failure patients received NIV between July and August 2017. There were 34 patients of NIV success (69.4%) and 15 patients of NIV failure (30.6%). The ratio of NIV success per NIV failure was 1: 2 STATA version 14.0 analysis software was used to calculate the sample size by employing a two-sample comparison of NIV success and NIV failure. The assumptions were as follows: alpha = 0.05 (two-sided test), power of sample size = 0.9, and the ratio of sample size = 1: 2 The sample size of 59 was obtained in NIV success population group and the sample size of 28 was obtained in NIV failure population group.

### Statistical analysis

Data were analyzed using STATA version 14.0. All study variables were compared between the NIV success (did not receive intubation) and the NIV failure (receive intubation) groups by using exact probability test for categorical study variables, and T-test in continuous study variables. The predictive power of each variable was calculated using univariable logistic regression and presented as the area under the receiver operating characteristic (AuROC) curve with 95% confidence intervals (CIs). The potential predictors were categorized into three levels by multivariable logistic regression. Regression coefficients for each level of each clinical predictor were divided by the smallest coefficient of the model and rounded to the nearest 0 or 0.5, resulting in a scoring scheme. Discrimination of the prediction scores was presented as the AuROC curve, and 95% CIs for the clinical scoring of failure on acute respiratory failure patients received NIV. Calibration of the prediction was presented using the Hosmer-Lemeshow goodness-of-fit test. The number of reports and percentages of each group were presented with the positive likelihood ratio, 95% CIs, and *p*-value.

## Results

**Fig. 1:**
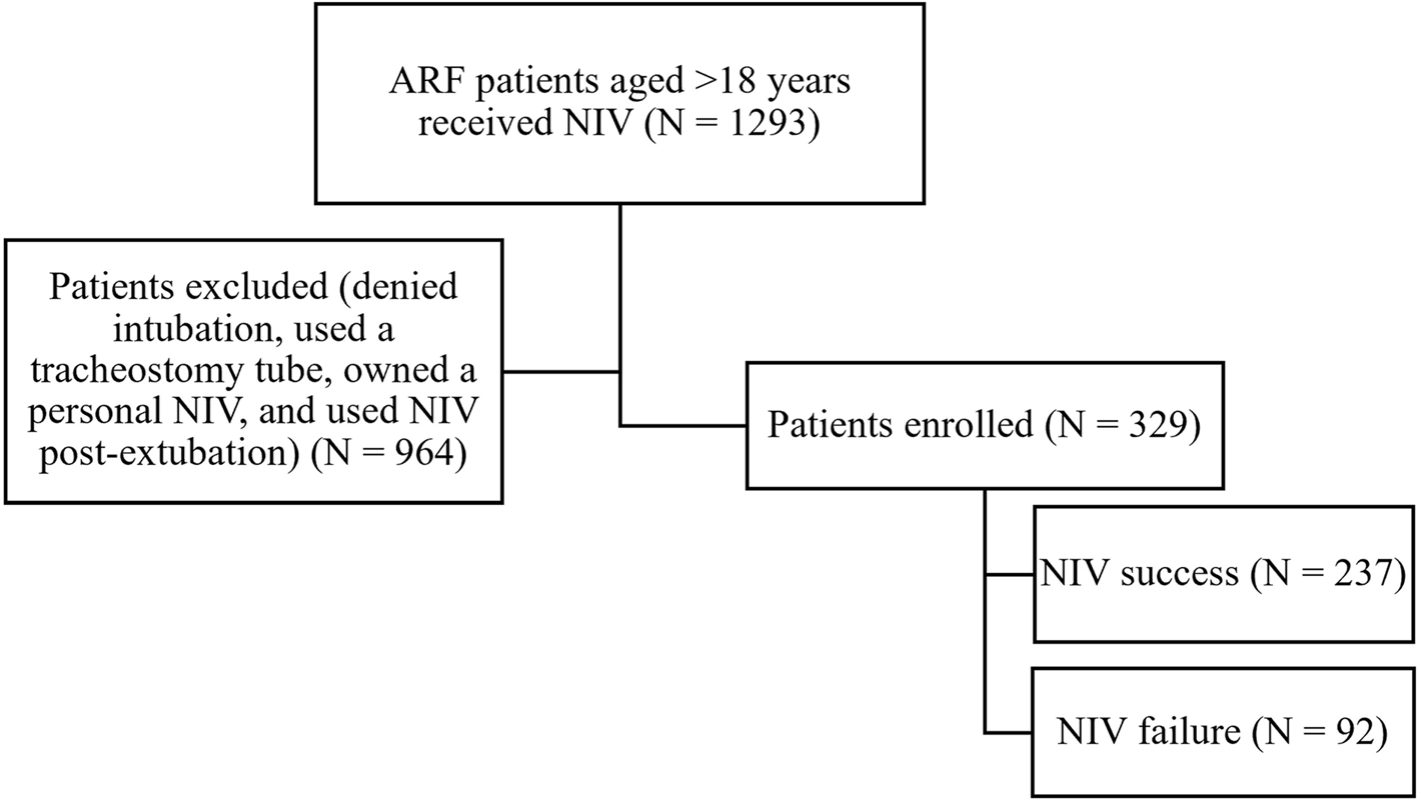
Flow of patients through the study. NIV = Non-invasive mechanical ventilation

The study variables of NIV success and NIV failure on acute respiratory failure patients was collected between February 2017 and December 2017 at the Emergency Medicine Department of Ramathibodi Hospital, a university-affiliated super tertiary care hospital in Bangkok, Thailand (Fig. 1). A total of 329 acute respiratory failure patients were received NIV success (*N* = 237) and failure (*N* = 92). As illustrated in Table 1, the acute respiratory failure patients possessed five variable factors including heart rate >  110 bpm, systolic BP <  110 mmHg, SpO2 < 90%, arterial pH < 7.30 and serum lactate significantly demonstrated the failure for receiving NIV, with high discriminative performance (*p* = 0.005, AuROC = 0.582; *p* = 0.001, AuROC = 0.564; p = 0.001, AuROC = 0.601; *p* = 0.016, AuROC = 0.526 and *p* <  0.001, AuROC = 0.589, respectively).

**Table 1:** The study variables of NIV success and NIV failure in acute respiratory failure patients

Baseline characteristics	Success (***N*** = 237)		Failure (***N*** = 92)		***p***-value	AuROC (95% CI)
**Gender**, Female (N, %)	130	54.85%	39	42.39%	0.049	0.438 (0.378–0.498)
**Age (years)**	74.78 ± 12.78		73.37 ± 13.79		0.382	–
Age > 75	150	63.29%	53	57.61%	0.377	0.472 (0.412–0.531)
**Vital signs at ED arrival**						
**Respiratory rate** (bpm)	29.46 ± 5.39		30.76 ± 6.88		0.070	–
Respiratory rate > 30	96	40.51%	52	56.52%	0.010	0.580 (0.520–0.640)
**Heart rate** (bpm)	97.82 ± 21.51		105.87 ± 26.67		0.005	–
Heart rate > 110	64	27.00%	40	43.48%	0.005	0.582 (0.524–0.641)
**Systolic BP** (mmHg)	156.68 ± 32.99		142.03 ± 39.27		< 0.001	–
SBP < 110	16	6.75%	18	19.57	0.001	0.564 (0.520–0.608)
**Oxygen saturation** (%)	92.31 ± 6.03		87.84 ± 11.21		< 0.001	
SpO_2_ < 90	55	23.21%	39	43.33%	0.001	0.601 (0.543–0.659)
**Body temperature** (°C)	37.13 ± 0.86		37.39 ± 0.90		0.015	
Body temp > 37.5	58	24.47	37	40.22	0.007	0.579 (0.521–0.636)
**Glasgow coma scale**						
GCS 13–15	229	96.62%	84	93.33%	0.222	0.517 (0.488–0.545)
GCS 9–12	8	3.38%	6	6.67%		
**Laboratory test**						
**White blood cell** (× 10^3^/μL)	9362.62 ± 4531.24		13,929.24 ± 15,127.59		< 0.001	–
WBC > 10,000	78	32.91%	53	57.61%	< 0.001	0.624 (0.565–0.682)
**Hemoglobin** (g/dL)	10.98 ± 2.16		11.51 ± 2.08		0.048	–
HgB < 12 in female or HgB < 13 in male	174	73.42%	68	73.91%	1.000	0.503 (0.449–0.556)
**Hematocrit** (%)	33.94 ± 7.07		35.03 ± 7.96		0.227	–
Hct < 36 in female or Hct < 40 in male	161	67.93%	55	59.78%	0.196	0.459 (0.401–0.518)
Blood urea nitrogen (mg/dL)(Med, IQR)	29.31 ± 21.82(22.00, 25.00)		33.84 ± 26.85(24.50, 32.00)		0.115	–
BUN > 18	142	59.92%	62	67.39%	0.255	0.537 (0.480–0.595)
**Creatinine** (mg/dL)	2.30 ± 2.77(1.28, 1.59)		2.19 ± 2.48(1.21, 1.55)		0.742	–
Cr > 1.02	146	61.60%	51	55.43%	0.318	0.469 (0.409–0.529)
**Sodium** (mEq/L)	136.45 ± 5.19		134.71 ± 6.71		0.013	–
136–145	147	62.23%	46	50.00%	0.090	0.558 (0.498–0.618)
< 136	86	36.29%	45	48.91%		
> 145	4	1.69%	1	1.09%		
**Potassium** (mEq/L)	4.23 ± 0.66		4.33 ± 0.92		0.275	–
3.5–5.1	169	71.31%	61	66.30%	0.247	0.532 (0.474–0.591)
< 3.5	41	17.30%	14	15.22%		
> 5.1	27	11.39%	17	18,48%		
**Arterial blood gas**						
**pH**	7.40 ± 0.04		7.40 ± 0.07		0.506	–
Acidosis < 7.30	3	1.27%	6	6.52%	0.016	0.526 (0.500–0.552)
**PaO**_**2**_ (mmHg)	121.70 ± 37.55		125.28 ± 59.73		0.517	–
PaO_2_ < 60	13	5.49%	7	7.61%	0.451	0.511 (0.480–0.541)
**PaCO**_**2**_ (mmHg)	39.44 ± 6.25		37.85 ± 11.28		0.106	–
PaCO_2_ > 50	11	4.64%	5	5.43%	0.778	0.504 (0.477–0.531)
**HCO**_**3**_ (mEq/L)	22.14 ± 4.14		20.53 ± 6.61		0.014	–
> 22	105	44.49%	30	33.33%	0.078	0.444 (0.386–0.503)
**Lactate** (mmol/L)	2.61 ± 1.03		3.50 ± 2.67		< 0.001	–
< 4	227	95.78	72	78.26	< 0.001	0.589 (0.544–0.633)
4–8	8	3.38	12	13.04		
> 8	2	0.84	8	8.70		
**FiO**_**2**_	0.26 ± 0.16		0.32 ± 0.23		0.017	–
PaO_2_ / FiO_2_ (mmHg)	531.67 ± 181.01		499.40 ± 229.79		0.181	–
PaO_2_ / FiO_2_ < 300	33	13.92%	20	21.74%	0.095	0.539 (0.491–0.587)
**Vasoactive agents** (N, %)	19	8.05%	16	17.58%	0.017	0.452 (0.409–0.495)
**qSOFA score** > 2	14	5.91%	16	17.78%	0.002	0.559 (0.517–0.602)
**Diagnosis (N, %)**						
Volume overload	32	13.50%	4	4.35%	0.017	0.546 (0.516–0.576)
Congestive heart failure	115	48.52%	29	31.52%	0.006	0.585 (0.528–0.642)
Tracheobronchitis	23	9.75%	11	11.96%	0.550	0.489 (0.451–0.527)
COPD	55	23.31%	23	25.00%	0.774	0.492 (0.439–0.544)
Pneumonia	63	26.58%	43	46.74%	0.001	0.399 (0.341–0.458)
Pleural effusion	15	6.33%	8	8.70%	0.473	0.488 (0.455–0.521)
Pulmonary embolism	4	1.69%	3	3.26%	0.404	0.492 (0.472–0.512)
ARDS	0	0.00%	5	5.43%	0.002	0.473 (0.450–0.496)
Sepsis	28	11.81%	23	25.00%	0.006	0.434 (0.385–0.483)
Septic shock	1	0.42%	16	17.39%	< 0.001	0.415 (0.376–0.454)
Anemia	16	6.75%	3	3.26%	0.297	0.518 (0.493–0.542)
Influenza	26	10.97%	15	16.30%	0.196	0.473 (0.430–0.516)
Neurological disease	1	0.42%	3	3.26%	0.068	0.486 (0.467–0.505)
Acute kidney injury	39	16.46%	31	33.70%	0.001	0.414 (0.360–0.468)
**Revisit in 7 days** (N, %)	15	6.33%	8	8.70%	0.473	0.488 (0.455–0.521)

The multivariable analysis showed item score of the significant predictors in the NIV failure including heart rate > 110 bpm (score = 0, 1), systolic BP < 110 mmHg (score = 0, 2), SpO2 < 90% (score = 0, 1), arterial pH < 7.30 (score = 0, 3) and serum lactate (score = 0, 2, 4) (Table 2).

**Table 2:** Significant predictors and item score of the NIV failure in acute respiratory failure patients

Predictors	Category	OR	95% CI	***p***-value	Coefficient^**a**^	Score
Heart rate > 110 bpm	No	1.00	reference	–	–	0
	Yes	1.84	1.05–3.22	0.033	0.61	1
Systolic BP < 110 mmHg	No	1.00	reference	–	–	0
	Yes	3.04	1.39–6.62	0.005	1.11	2
SpO_2_ < 90%	No	1.00	reference	–	–	0
	Yes	2.44	1.40–4.26	0.002	0.89	1
Arterial pH < 7.30	No	1.00	reference	–	–	0
	Yes	4.94	1.01–24.01	0.048	1.60	3
Serum lactate (mmol/L)	< 4	1.00	reference	–	–	0
	4–8	3.09	1.13–8.47	0.029	1.13	2
	> 8	12.22	2.38–62.68	0.003	2.50	4

^a^Coefficients from multivariable continuation ratio logistic regression

*OR* odds ratio; *CI* confidence interval; *bpm* beat per minute; *BP* blood pressure; *SpO*_*2*_ Pulse oxygen saturation

**Fig. 2:**
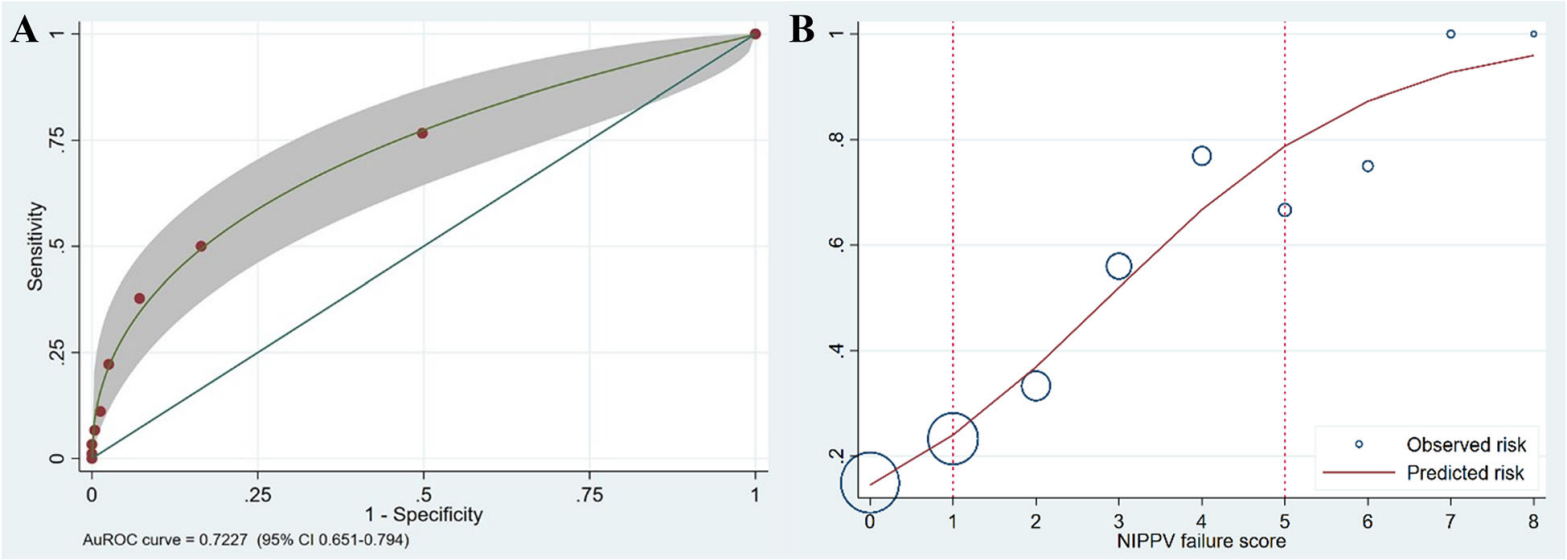
The AuROC and 95% Confidence Interval of the predictive power of the clinical scoring (**a**) and Observed risk (circles) vs score-predicted risk (solid line) (**b**) of the NIV failure in acute respiratory failure patients

As shown in Fig. 2, this study exhibited that the AuROC curve was 72. 27% (95% CI: 0.651–0.794) for the ability of the clinical score to predict the failure of NIV, and the increased score-predicted risk correlated to the observed risk of the failure of NIV in acute respiratory failure patients. The clinical scoring of the NIV failure in acute respiratory failure patients was classified into three groups: low, score 0–1; moderate, score 2–4; and high, score >  5. The positive likelihood ratio in the high group was 8.78 (Table 3). The patient should undergo intubation or definite airway instead of NIV.

**Table 3:** Distribution of NIV failure vs NIV success into low, moderate and high probability categories, likelihood ratio of positive (LHR+) and 95% confidence interval (CI)

Probability categories	Score	Failure ***N*** = 92		Success ***N*** = 237		LHR+	95% CI	***p***-value
		N	%	N	%			
Low	0–1	45	50.00	198	83.54	0.60	0.48–0.74	< 0.001
Moderate	2–4	35	38.89	36	15.19	1.70	1.15–2.50	0.006
High	> 5	10	11.11	3	1.27	8.78	2.47–31.17	< 0.001
Mean ± SD		0.77 ± 1.04		2.09 ± 1.93				< 0.001

## Discussion

Non-invasive mechanical ventilation (NIV) is an alternative therapy to avoid the life-threatening risks of invasive mechanical ventilation. It uses ventilatory support via the patient’s upper airway using a mask or similar device [3, 10]. Absolute contraindications of NIV contain cardiorespiratory arrest, extreme psychomotor agitation, severe hemodynamic instability, non-hypercapnic coma, and multiple organ failure [3]. NIV is a widely used and effective treatment for acute respiratory failure (ARF), particularly an acute exacerbation of chronic obstructive pulmonary disease (COPD) and cardiogenic pulmonary edema since the 1980s [11]. Previous studies demonstrated high success and low mortality rates of NIV in patients [12]. However, NIV failure may delay intubation, which may increase mortality and health care costs. Various clinical scoring strategies of NIV failure were assessed for early prediction. Five variables, including heart rate, acidosis, consciousness, oxygenation, and respiratory rate (HACOR) scores showed good predictive power for NIV failure in COPD patients, particularly for the prediction of early NIV failure (< 48 h) [13].

Due to the lack of the best clinical scoring, this study assessed the NIV failure patients’ novel clinical scoring in acute respiratory failure patients at the emergency department. This study showed that NIV failure was associated with heart rate > 110 bpm, systolic BP < 110 mmHg, SpO2 < 90%, arterial pH < 7.30 and serum lactate. The clinical scores were classified into three groups: low, moderate, and high. We suggested that the low group increased the chance of successful NIV in ARF.

In this study, serum lactate levels (> 8 mmol/L) were the most relevant variables for predicting NIV failure, with a maximal score of 4 points. Blood lactate was considered a diagnostic hallmark of tissue hypoxia, respiratory muscle fatigue, and COPD severity [14]. Patients with a high lactate level are strongly correlated with increased mortality in various conditions [15–17]. Arterial pH (< 7.30) was the second most relevant variable, with a maximal score of 3 points, followed by systolic BP (< 110 mmHg) with a maximal score of 2 points. The pH level, an indicator of hypercapnia severity, has been documented as an essential predictor to assess NIV success [18].

Previous studies have been clearly reported that a lower baseline pH is a risk factor for NIV failure in certain conditions, especially in COPD patients [19, 20]. COPD patients with mild to moderate acidosis showed that NIV improved patient outcomes exclusively, the baseline pH was ≥7.30 [21]. In a certain study, systolic BP < 90 mmHg is considered a relative contraindication to NIV [22]. Heart rate (> 110 bpm) and SpO2 (< 90%) were less relevant, with a maximal score of 1 point. Generally, a heart rate < 110 bpm has been suggested as an indicator to withdrawn NIV in ARF [10]. SpO2, an arterial oxygen saturation measured by pulse oximetry, targets should be 88–92% in hypercapnic ARF patients treated with NIV. A previous study demonstrated that no single variable could predict NIV failure well. On the other hand, a combination of several variables may increase predictive accuracy [13].

There are limitations to this study. First, this study was retrospective data collection and conducted in a single center. The clinical parameters are affected by confounder variables, overlapping that can incorporate normal parameters. Some parameters are poor predictors—the predictor variables in other studies not statistically significant in this study. We now need to validate our results externally to establish our risk score’s actual value for management decisions.

## Conclusion

Using combination of 5 variables including heart rate > 110 bpm, systolic BP < 110 mmHg, SpO2 < 90%, arterial pH < 7.30 and serum lactate. The clinical scores were classified into three groups: low, moderate, and high.

## Data Availability

The datasets analysed during the current study are not publicly available due to privacy issues but are available from the corresponding author upon reasonable request.

## Abbreviations

NIV: Non-invasive mechanical ventilation
ARF: Acute respiratory failure
AuROC: Area under the receiver operating characteristic curve
CIs: Confidence intervals
COPD: Chronic obstructive pulmonary disease
